# SNAP25 Inhibits Glioma Progression by Regulating Synapse Plasticity *via* GLS-Mediated Glutaminolysis

**DOI:** 10.3389/fonc.2021.698835

**Published:** 2021-08-16

**Authors:** Qiongzhen Huang, Changlin Lian, Yaoyuan Dong, Huijun Zeng, Boyang Liu, Ningbo Xu, Zhenyan He, Hongbo Guo

**Affiliations:** Guangdong Provincial Key Laboratory on Brain Function Repair and Regeneration, Zhujiang Hospital, Department of Neurosurgery, Guangzhou, China

**Keywords:** SNAP25, synaptic plasticity, glutaminase, glioma progression, glutamine metabolism

## Abstract

**Background:**

Neuronal activity regulated by synaptic communication exerts an important role in tumorigenesis and progression in brain tumors. Genes for soluble N-ethylmaleimide-sensitive factor attachment protein receptors (SNAREs) annotated with the function ‘vesicle’ about synaptic connectivity were identified, and synaptosomal-associated protein 25 (SNAP25), one of those proteins, was found to have discrepant expression levels in neuropathies. However, the specific mechanism and prognostic value of SNAP25 during glioma progression remain unclear.

**Methods:**

Using RNA sequencing data from The Cancer Genome Atlas (TCGA) database, the differential synaptosis-related genes between low grade glioma (LGG) and glioblastoma (GBM) were identified as highly correlated. Cox proportional hazards regression analysis and survival analysis were used to differentiate the outcome of low- and high-risk patients, and the Chinese Glioma Genome Atlas (CGGA) cohort was used for validation of the data set. RT-qPCR, western blot, and immunohistochemistry assays were performed to examine the expression level of SNAP25 in glioma cells and samples. Functional assays were performed to identify the effects of SNAP25 knockdown and overexpression on cell viability, migration, and invasion. Liquid chromatography-high resolution mass spectrometry (LC-MS)-based metabolomics approach was presented for identifying crucial metabolic disturbances in glioma cells. In situ mouse xenograft model was used to investigate the role of SNAP25 *in vivo*. Then, an immunofluorescence assay of the xenograft tissue was applied to evaluate the expression of the neuronal dendron formation marker-Microtubule Associated Protein 2 (MAP2).

**Results:**

SNAP25 was decreased in level of expression in glioma tissues and cell lines, and low-level SNAP25 indicated an unfavorable prognosis of glioma patients. SNAP25 inhibited cell proliferation, migration, invasion and fostered glutamine metabolism of glioma cells, exerting a tumor suppressor role. Overexpressed SNAP25 exerted a lower expression level of MAP2, indicating poor neuronal plasticity and connectivity. SNAP25 could regulate glutaminase (GLS)-mediated glutaminolysis, and GLS knockdown could rescue the anti-tumor effect of SNAP25 in glioma cells. Moreover, upregulated SNAP25 also decreased tumor volume and prolonged the overall survival (OS) of the xenograft mouse.

**Conclusion:**

SNAP25, a tumor suppressor inhibited carcinogenesis of glioma *via* limiting glutamate metabolism by regulating GLS expression, as well as inhibiting dendritic formation, which could be considered as a novel molecular therapeutic target for glioma.

## Introduction

Gliomas are among the most common primary brain tumors in adults and account for over 70% of malignant brain tumors, of which glioblastoma (GBM) is the most aggressive and deadly type with a median survival of 15 months and 5-year overall survival of 5.5% (1). Despite the conventional treatments (surgery followed by chemotherapy and radiotherapy), the prognosis of GBM has not been improved over the past years due to the highly invasive residual tumor cells and the incompletely resected tumors. Resistance of malignant gliomas to conventional therapies has been widely reported as a consequence of oncogene signaling activation and distinct metabolic mechanisms when cancer cells are exposed to various chemotherapeutic and/or cytostatic agents, thus, recurrent tumors usually become more aggressive (2, 3). Thus, the specific mechanism of glioma progression needs to be identified.

Previous studies have confirmed that gliomas occur in a striking spatiotemporal pattern highlighting the critical importance of the tumor microenvironment (4), as close relationships between glioma cells and neighboring microglia, astrocytes, and vascular cells have recently come to light (5, 6). Microenvironmental interaction, especially the aberrant interplay between glia and synapses, have been posted to contribute to neural pathology of Rett syndrome (7), Down syndrome (8), Spinal Muscular Atrophy (9) and others (10). More recent studies have identified that the communication between neurons and glial cells is associated with several neuropsychiatric and neurodegenerative disorders such as schizophrenia (11). Glias are active participants in synaptic plasticity and are known to modulate individual synapses and circuits (12). Importantly, the function of astrocytic glutamate transporters GLT-1 and GLAST is a classic example of how astrocytes regulate glutamatergic synaptic transmission by controlling the neurotransmitter levels at the synapse (13). Glutamate not only participates in synapse communication as one of the functional neurotransmitters but also functions as a signature metabolic product in tumor origination (14). Cancer cells typically rewire their metabolism to meet the bioenergetic and biosynthetic demands of uncontrolled cell growth, for example, many oncogenic mutations result in enhanced glutamate metabolism, reflecting its importance on tumor progression by generating tricarboxylic acid (TCA) cycle intermediates and amino acids, and maintaining redox homeostasis (15, 16). In this context, the rate-limiting enzyme converting glutamine to glutamate, GLS, primarily enhances glutaminolysis and may provide a typical target in glutamate metabolism pathways (17). Therefore, targeting glutamate metabolism is an appealing therapeutic option in many cancer subtypes.

SNAP25 is a member of the SNARE family, associated with severe synaptopathies like Schizophrenia and also proteinopathies like Alzheimer’s disease but its function in glioma is seldom studied (18–20). A recent study indicated that SNAP25 was a microenvironment-related gene that predicted poor outcomes in colon cancer, and gene set enrichment analysis (GSEA) suggested that SNAP25 was involved in metabolism progress (21).

In this study, SNAP25 was mined to have significantly lower expression levels in glioma from the mRNAs expression profiles in TCGA dataset and lower-expressed SNAP25 indicated an unfavorable prognosis of glioma patients. Then the lower expression of SNAP25 in glioma tissues and cell lines was validated. The effect of SNAP25 on glioma progression was studied, and the underlying metabolic and synaptic plasticity by which SNAP25 regulated glioma cell phenotype was also investigated.

## Materials and Methods

### Datasets

We collected 693 and 512 gliomas with RNA‐seq data and clinical information from TCGA and CGGA databases, respectively. All tissues and clinicopathologic information were obtained with written informed consents.

### Screening DEGs Through Integrated Analysis

The background correction, standardization, and log 2 conversion for raw data were conducted by the “affy” package of Bioconductor according to the annotation files. We used the “limma” package of the R software to investigate DEGs in LGG and GBM tissues. *P*-value < 0.05 along with |logFC| > 1 were considered significant.

### Patients and Specimens

All 40 glioma tissues samples and 8 normal samples were obtained from patients who had received surgery and chemotherapy at Zhujiang Hospital (Southern Medical University, Guangzhou, China). These glioma specimens include 16 Grade IV (GBM), 14 Grade III, 8 Grade II, and 2 Grade I astrocytoma cases, and the histologic features of surgical resection specimens were independently examined by two neuropathologists according to the WHO criteria (**Supplementary Table 1** **)**. The specimens were frozen in liquid nitrogen immediately after the surgery and then paraffin embedded for long time preservation. The project protocol was approved by the Ethics Committee of Zhujiang Hospital and written informed consents were obtained from all patients enrolled in this study.

### Cell Culture

The human GBM cell lines (U87, U251, A172, U118) were purchased from the Cell Bank of the Chinese Academy of Sciences (Shanghai, China), and were authenticated and examined for mycoplasma contamination. NHA cells were kindly provided by the Yongping You’s lab of Nanjing medical university. U87MG-mCherry(U87MC) glioma cell line labelled with red fluorescent protein were kindly provided by the Ke Yichuan’s lab of South medical university. The cells were routinely cultivated at 37°C in the 5% CO_2_ humidification incubator (Thermo Scientific, Waltham, MA, USA) in Dulbecco’s modified Eagle’s medium (Invitrogen) with fetal bovine serum (10% v/v, Hyclone, Logan, UT, USA) penicillin (200 units/ml) and streptomycin (100 μg/ml).

### Cell Lentiviral Transfection

For lentiviral transfection, cells were seeded at 50% confluence in six-well cell culture plates and incubated with 1 ml medium overnight. Then medium was replaced with 500ul micture of OPTI-MEM (Invitrogen, USA) with polybrene (4 ug/ml, Genechem, Shanghai, China). Cells were transfected by adding control shRNA lentiviral vectors, SNAP25 shRNA lentiviral vectors, GLS shRNA lentiviral vectors, Control lentiviral activation vectors, SNAP25 lentiviral activation vectors, GLS lentiviral activation vectors, respectively. All the lentiviral vectors were obtained from Obio (Obio, Shanghai, China). Medium was replaced with complete medium without polybrene 24h later after transfection. Transduced cells were selected for puromycin (2ug/ml, sc-108071, Santa Cruz) resistance for 10 days. The gene expression efficiency was detected by qRT-PCR and western blot.

### RNA Isolation, Reverse Transcription, and Quantitative Real-Time PCR

Total RNA from specimens or cells was extracted by using Trizol Reagent (Takara Bio, Shiga, Japan) and the absorbance was measured with OD260/280 ratio higher than 1.8. For qPCR analysis, cDNA was synthesized with the Prime ScriptTM RT reagent (Takara Bio, Shiga, Japan). Quantitative real-time PCR assay by using SYBR GREEN PCR Master Mix (Takara Bio, Shiga, Japan) was performed in triplicate with GAPDH as endogenous controls and the gene expression relative to control was calculated by 2^−ΔΔCT^.

### Western Blot Assay

Total protein of cells was extracted by Cell Lysis and Protein Extraction kit (Keygen Biotech Co., China) and the western blot was performed in standard procedures. Specific antibodies were applied to the western blot: SNAP25(1:1000; Rat# ab5666;Abcam), Glutaminase(1:1000; Rat# ab156876;Abam), β-catenin (1:1000; Rat#3700S; Cell Signaling Technology). Subsequently, the blots were incubated with goat anti-rabbit or mouse IgG (H+L) secondary antibody (Fdbio, China) at room temperature for 2 h. Then the blots were washed with TBST and visualized. The analysis of the protein expression was performed by the Image J software with β-actin as endogenous controls, and then the image is drawn according to the gray value using the software graphpad.

### Immunohistochemistry

The paraffin tissue was sliced continuously with a thickness of 4 microns and dried in an oven at 68°C, the slices were then dewaxed in three different concentrations of xylene solutions for 10 minutes each, then were placed in 100%, 95%, 85%, 75% alcohol for hydration, for 5 minutes each. The hydrated slices were rinsed slowly under running water for ten minutes and then dried. Fifty microliters of 3% hydrogen peroxide solution were added to each section, and the tissue on the section was evenly covered and incubated at room temperature for 15 minutes to seal, the slices were rinsed in running water for 10 minutes in the manner previously described, the antigenic repair solution was dropped into the rinsed section and then heated at 95°C to 99°C for 20 minutes. After the slices were naturally dried, they were rinsed twice with PBS buffer for 5 minutes each time, after the slices were dried again, a drop of 5% goat serum solution was added evenly to each slice, and the slices were incubated at room temperature for 30 minutes, then the serum was removed. 10 microliters anti-SNAP25 antibody (1:400; Rat# ab5666; Abcam) or anti-GLS antibody (1:400; Rat# ab156876;Abam) was added to 1 mL PBS and diluted evenly, then the reagent is dripped onto the section at 4°C overnight, the next day, the sections were left at room temperature for 30 minutes and rinsed with PBS 3 times for 3 minutes each time. Then sections were incubated with secondary antibody at 37°C for 1 h, then rinsed three time with PBS buffer for 3 minutes each time. DAB colorimetric solution is added to the slices, when the chromogenation is observed under a microscope, the sections are washed with running water. The sections were stained with hematoxylin for 3 minutes, washed with running water for 3 minutes, differentiated in 1% alcohol for 1-3 seconds, washed with running water, placed in PBS, then washed with running water, and placed in 75%, 85%, 95%, 100% alcohol for dehydration, each dehydration for 5 minutes. The dehydrated sections were placed in a solution of xylene to make them transparent and then dried. Each section was dripped with 20 microliters of neutral resin and covered with cover glasses, sections were examined microscopically for staining.

### Immunofluorescence Staining

Glioma cells were respectively positioned on glass coverslips (0.17 mm thickness, 14 mm diameter) in a 6-well plate at room temperature overnight. Then cells were washed by PBS, fixed by 4% paraformaldehyde for 30min, infiltrated by 0.1% Triton X-100 for 5min, and blocked by 2% bovine serum albumin (BSA) for 30min in sequence. Incubated with Specific primary antibodies: anti-SNAP25 (1:400; Rat# ab5666;Abcam), anti-GLS (1:400; Rat# ab156876;Abam), anti-MAP2 (1:400;Rat# ab5392; Abcam) at 4°C overnight and rinsed by PBS 3 times, fluorescent secondary antibodies (Donkey anti-Rabbit IgG (H+L) Highly Cross-Absorbed Secondary Antibody, Alexa Fluor 488 (Thermo Fisher Scientific, catalog# A-21206, RRID AB_2535792) were applied to specimens and incubated at 37°C in the darkness for 1h. Mounting medium with DAPI DNA counterstain was applied to the specimens followed by images capture (Nikon, Ti2-E).

### Tumor Xenograft Model

For the murine Intracranial tumors generation, 5×10^5^ specified cells (U87MC NC- SNAP25, U87MC oe-SNAP25, U87MC oe NC- GLS and U87MC oe sh05-GLS cells) expressing luciferase were independently injected into the randomly grouped mice and the bioluminescence was examined on the 0^th^, 7^th^, 14^th^, 21^th^ day. Then the brains were dissected for immunohistochemistry and immunofluorescence staining. Procedures in experiments were performed according to the National Institutes of Health Guide for the Care and Use of Laboratory and approved by the Animal Experimental Committee of Southern Medical University.

### CCK-8 Assay

The cells were seeded in a 96-well plate for 24 h after stable transfection or transient transfection. Then followed by incubating with 10% CCK-8 (Dojindo, Japan) solution fresh medium solution for 2 h, the absorbance was measured at 450 nm using Ultra Multifunctional Microplate Reader (Tecan, Switzerland) according to the instructions.

### Wound Healing

The cells were cultured in a 6-well plate, scraped with cells to draw a line in the central area after the cells were scraped, and the cells in this line were mechanically removed. Then the cells were continued to be cultured in a serum-free medium, and the migration of cells to the scratch area without cells was observed to judge the migration ability of the cells.

### Transwell Migration Assay

The invasive abilities of cells were assessed through the transwell inserts (353097-Falcon, BD) with a 1:4 diluted Matrigel coating layer. After being suspended in a serum-free medium, the tumor cells were seeded into the upper well of the chamber for 24h and a medium with 10% FBS was supplied in The lower well. Then in the filter, the cells on the upper surface were removed and the cells on the lower surface were stained with 1% crystal violet after treated with 4% paraformaldehyde. The number of cells were calculated in 9 random fields using the microscope (×400).

### Cancer Cell Spheroid Invasive Assay

Tumor cell invasion was assessed using a three-dimensional (3D) spheroid invasion assay (22). U118 and A172 cells formed spheres in hanging drops of culture medium on the lid of cell culture dishes (approximately 500 cells per drop). After 48 hours, spheres from the lid were aliquoted into the same volume, mixed with rat tail type I collagen (final concentration is 1.7mg/ml), and embedded in wells to generate a 3D culture system. The invasion was concluded at 48 hours. Quantitative analyses were determined by measuring the maximal invasive distance (longest invasive distancespheroid radium) and invaded area (total invaded area-spheroid area) using the Image J software.

### LC-MS Analysis

For LC-MS analysis, in analytical triplicate, 5 μL of the sample was injected on an XBridge BEH amide column through an Acquity H-class UPLC system (Waters Corporation). MS was done using a Waters Xevo-TQS-micro MS with polarity-switching (positive mode 3 kV, negative mode 2 kV), and multiple reaction monitoring modes were used to acquire data with a randomized injection order. Before, during, and after the run, quality control (QC) samples were injected. Data were processed through TargetLynx (v4.1) to identify peaks from Total Intensity Chromatograms. Peaks were then integrated, and ion counts were obtained and exported for further processing in R. Metabolites found in < 50% QC samples or those with a coefficient of variation > 30% were dropped. Besides, QC samples were used to fit a cross-validated locally estimated scatterplot smoothing (LOESS) function to each metabolite. This accounted for instrumental drift and was used for ion count normalization. The raw data of the LC-MS analysis was uploaded in Metabolights (https://www.ebi.ac.uk/metabolights/index) and the study number is MTBLS2806.

### Quantification and Statistical Analysis

All statistical analyses were conducted using GraphPad Prism 8.0. Data represent mean ± s.e.m. unless otherwise noted and reported as biological replicates with technical replicates specified in figure legends. Unpaired two-tailed Student t-tests were used to determine *p*-values. Significance was defined as *p < 0.05, **p < 0.005, and ***p < 0.0005.

## Results

### A Screen of Candidate Genes Associated With Tumor Progression and Prognostic Validity of the Candidate Gene for Glioma

**Figure 1:**
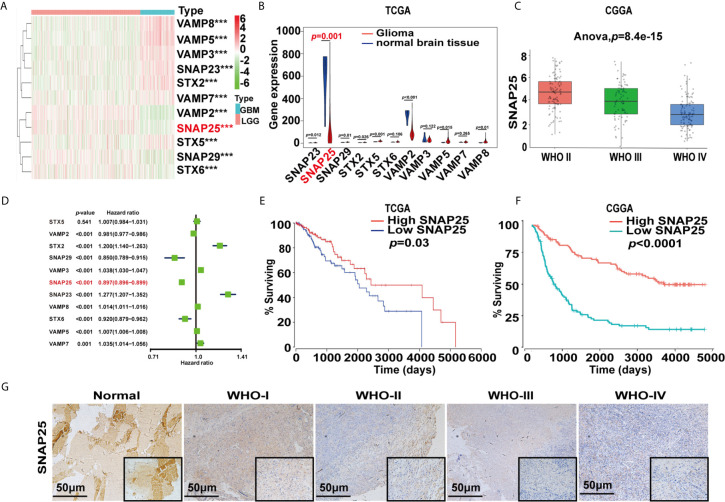
SNARE-related genes expression profiles and correlation between gene expression and clinical features in TCGA and CGGA datasets. **(A)** Heatmap show the different expression levels of 11 SNARE-related genes in TCGA dataset. Up-regulation is shown in red and downregulation is in blue. ***p < 0.001. **(B)** Violin plot shows the expression of SNARE-related genes in normal tissue and gliomas. **(C)** Box plot shows SNAP25 expression in different glioma grades in CGGA database. **(D)** Hazard ratio values of the eleven selected genes. **(E, F)** Kaplan-Meier survival analysis for glioma patients with low and high risk scores in TCGA and CGGA datasets. Kaplan-Meier survival curve for glioma patients with a high risk score (red line) and a low risk score (blue line). **(G)** Representative images of SNAP25 expression from glioma tissues and nontumor tissues by ISH assays.

To identify the key genes involved in tumor progression of glioma, 527 cases of LGG patients and 166 GBM cases from the TCGA database were selected to perform differential expression analysis. As described in *Materials and Methods*, all cases were divided into two groups: the “LGG” group and the “GBM” group. A total of 24,991 differentially expressed genes (DEGs) were significantly upregulated and downregulated in LGG samples and GBM samples using adjusted *p*<0.05 and |logFC|≥1 as the cut-off. Gene clustering using the R package ‘pheatmap’ found that the profile of synaptosome-related (SNARE) genes between LGG and GBM showed obvious differences and SNAP25 was significantly down-expressed in GBM (**Figure 1A**). Violin plot showed the exact changing level of these genes as SNAP25 expression in glioma was significantly lower than that in normal brain tissue (*p*=0.001, **Figure 1B**). Next, the CGGA dataset was selected to show a negative correlation between the expression of SNAP25 and the WHO grades of glioma (**Figure 1C**). By performing univariate Cox regression analyses to determine the prognostic value of the acquired gene set, SNAP25 was indicated to be independently correlated with OS (*p*<0.001, **Figure 1D**). Then, based on the median SNAP25 expression score, patients were assigned to the high- or low-SNAP25 expression group. Kaplan-Meier analysis found the low-SNAP25 cases had a significantly shorter OS than high-SNAP25 ones (*p*=0.03, **Figure 1E**). To validate this, we also calculated patients’ risk scores of the CGGA cohort. As expected, we acquired a consensus result (**Figure 1F**). Then, immunohistochemistry assay was applied to analyze the SNAP25 expression in surgical resection specimens, from which SNAP25 expression was negatively correlated with the tumor grading (*p*<0.001, Mann–Whitney test) (**Figure 1G**). Taken together, these results suggest that SNAP25 downregulation is associated with poor clinical outcomes of glioma.

### Association of SNAP25 With Glioma Cell Proliferation

**Figure 2:**
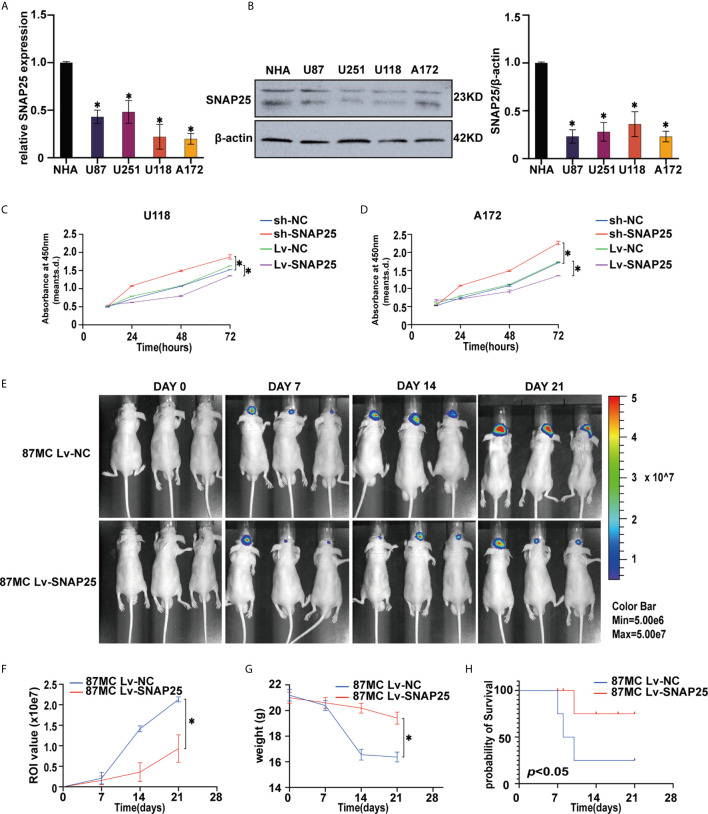
Overexpression of SNAO25 inhibits glioma cell growth in *vitro* and in *vivo*. **(A)** Relative SNAP25 expression levels in various glioma cell lines, compared with the normal human astrocytes (NHA), *p < 0.05 compared with NHA cells. GAPDH was used as a housekeeping gene. **(B)** The level of SNAP25 protein was detected in 4 glioma cell lines and NHA by western blot. *p < 0.05 compared with NHA cells. B-actin was used as a control and the barplot besides shows the representative SNAP25/actin ratio regarding to the western blot. **(C, D)** Ectopic expression of SNAP25 inhibits cell growth as determined by CCK-8 assay. *p < 0.05 compared with Lv-NC or sh-NC group. **(E)** Tumor growth was monitored over time (up to Day 21) by measuring luciferase emission (ROI). **(F–H)** The ROI value, weight of the mice and the probability of survival was recorded over time.

After we found a lower expression of SNAP25 in glioma, we explored its functional effects on glioma cells. First, we detected the expression of SNAP25 in glioma cell lines (U87, U251, U118, and A172), when compared to normal human astrocytes (NHAs) by RT-qPCR (**Figures 2A**) and western blot assays (**Figures 2B**). SNAP25 was decreased in level of expression in glioma cells, especially in U118 and A172 cells (**Figure 2A**). Therefore, we transfected U118, A172 cells with an shRNA targeting SNAP25 (U118-sh-SNAP25, A172-sh-SNAP25) and transfected them with functional SNAP25-cDNA (U118-Lv-SNAP25, A172-Lv-SNAP25). RT-qPCR and western blot assays confirmed that the expression of SNAP25 was effectively modulated in U118 and A172 cells (**Supplementary Figures 1A, B**).

Functionally, the CCK-8 results showed that overexpression of SNAP25 significantly decreased cell proliferation in U118 and A172 cells, whereas knockdown of SNAP25 significantly increased cell proliferation in U118 and A172 cells (**Figures 2C, D** **)**. Moreover, the effect of SNAP25 overexpression on tumor growth was also examined by a nude-mouse transplanted tumor model. The results exhibited that U87Mcherry Lv-SNAP25 delayed tumor growth, decreased the weight-loss of vehicle mice, and prolonged the survival time compared with the U87Mcherry Lv-NC group (**Figures 2E–H**).

In summary, these results demonstrated that SNAP25 could significantly inhibit glioma cell proliferation *in vitro* and sponge tumor growth *in vivo*.

### Relationship Between SNAP25 and Glioma Cell Migration, Invasion, and Dendritic Formation

**Figure 3:**
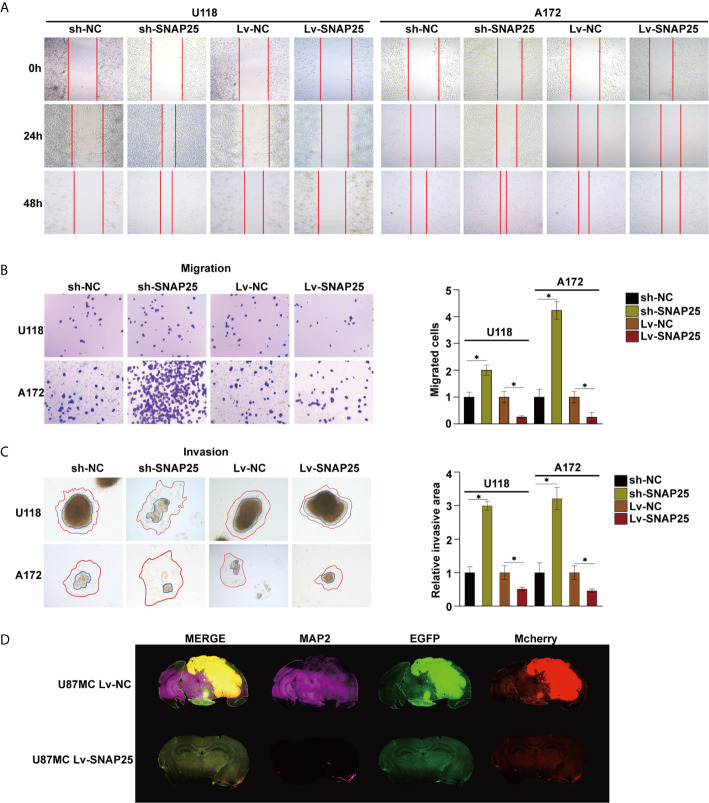
SNAP25 inhibits migration and invasion of tumor cells, and inhibits dendron formation of glioma tissues. **(A)** Ectopic expression of SNAP25 inhibits cell migration as determined by wound healing assays. **(B)** Ectopic expression of SNAP25 inhibits cell migration as determined by transwell assays and analysis of representative migrated rates of glioma cells is shown below. *p < 0.05 compared with Lv-NC or sh-NC group. **(C)** Representative pictures of glioma cell invasion (red curved line: invaded area, blue circle: spheroid) and analysis of quantitative invaded area of glioma cells using Image J software is shown below. *p < 0.05 compared with Lv-NC or sh-NC group. **(D)** Glioma cell (Mcherry staining) and neuronal dendron (MAP2 staining) in the brains of indicated mice at the time of harvesting (Day 21).

Subsequently, we examined the role of SNAP25 in glioma migration and invasion. The wound-healing assay indicated that the motility of gliomas cells with stable SNAP25 silencing was significantly increased, while in SNAP25-upregulated cells was decreased (*p*<0.05, **Figure 3A**). Transwell assays were implemented to evaluate the migration ability of glioma cells, and reduced migration ability of U118 and A172 cells with stable SNAP25 overexpression was observed (*p*<0.05, **Figure 3B**). Then we evaluated the effect of SNAP25 on tumor cell invasion using a 3-dimensional spheroid assay (22). Knockdown of SNAP25 increased invaded area and distance of both U118 and A172 cells (*p*<0.05, **Figure 3C**). As SNAP25 acts as a classic role in synapse formation and transmission, we examined the roles of SNAP25 in neuron-glioma cells dendritic processes by immunofluorescence staining of MAP2, a neuron-specific cytoskeletal protein enriched in dendrites and perikarya, which implicates a biomarker of neuron development. As shown in the immunofluorescence staining results of the glioma xenograft in **Figure 3D**, U87Mcherry Lv-SNAP25 glioma cell-transplanted-xenograft expressed lower MAP2 expression than U87Mcherry Lv-NC group, indicating a negative function of SNAP25 in synaptic plasticity. In general, SNAP25 overexpression inhibited cell migration and invasion of U118 and A172 cells, and SNAP25 knockdown promoted cell migration and invasion of glioma cells, and upregulation of SNAP25 could also inhibit the dendritic formation of the tumor *in vivo*.

### SNAP25 Activates GLS Expression in Glioma Cells

**Figure 4:**
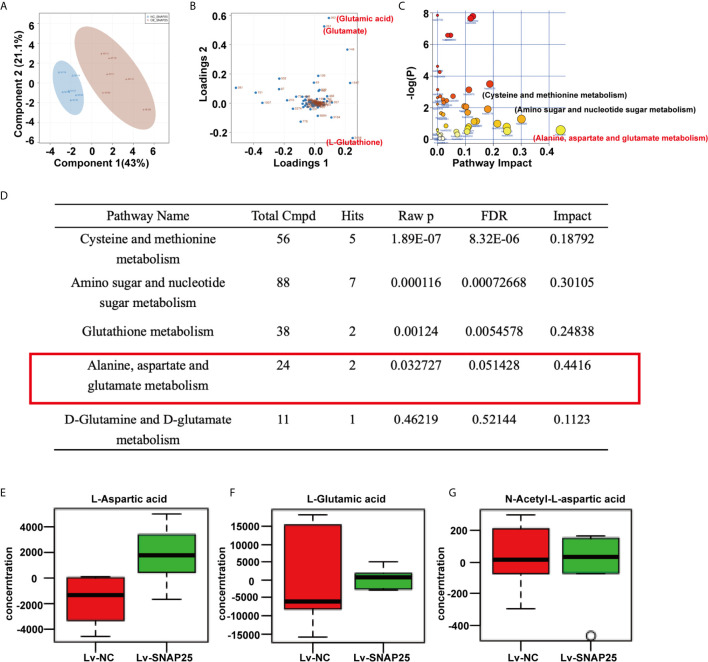
SNAP25 regulates glutamine metabolism of glioma cells. **(A)** Plots of the principle component analysis results for LC-MS data obtained for Lv-NC and Lv-SNAP25 glioma cells. **(B)** Loading plots of the principle component analysis results for LC-MS data obtained for Lv-NC and Lv-SNAP25 glioma cells. Metabolites 261 (glutamate), 262 (glutamic acid), and 2458 (L-glutathione) contributed largely to their separation. **(C, D)** Pathway analysis results for LC-MS data obtained for Lv-NC and Lv-SNAP25 glioma cells. Alanine, aspartate and glutamate metabolism pathway shows a high pathway impact (0.4416), *p*=0.03. **(E–G)** The concentration of L-Aspartic acid **(E)**, L-Gluatamic acid **(F)**, and N-Acetyl-L-aspartic acid.

**Figure 5:**
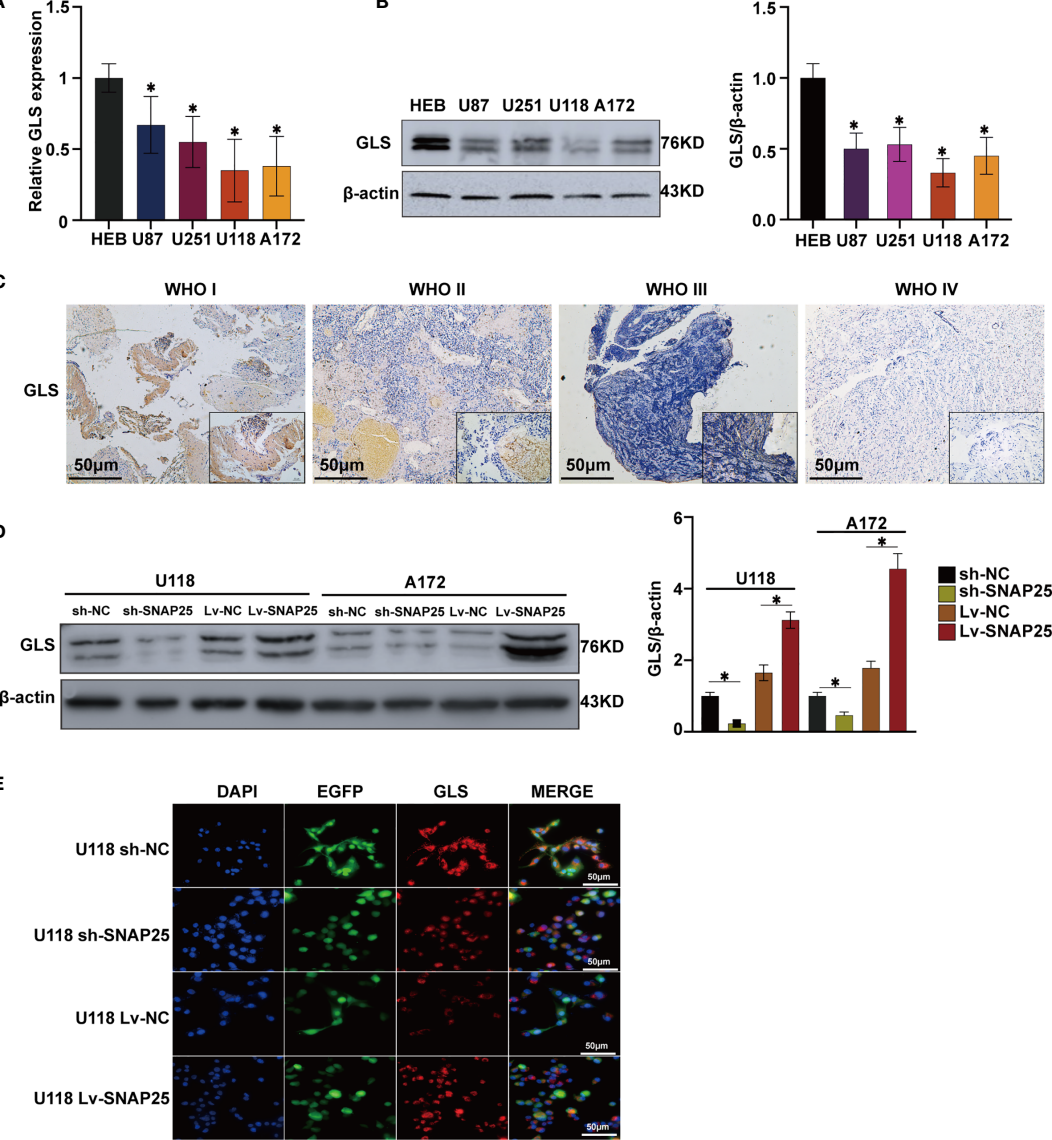
SNAP25 functions as an efficient sponge in glioma. **(A, B)** RT-qPCR **(A)** and western blot **(B)** analysis of GLS expression level in glioma parental cell lines and NHA, *p < 0.05 compared with NHA cells. **(C)** Representative images of SNAP25 expression from glioma tissues and nontumor tissues by ISH assays. **(D)**–Western blot analysis of GLS expression level in U118 and A172 glioma cell lines transfected with sh-NC, sh-SNAP25, Lv-NC and Lv-SNAP25, *p < 0.05 compared with Lv-NC or sh-NC group. **(E)** Immunofluorescence staining of GLS in SNAP25-transfected cells.

Metabolomic analysis was performed on A172 sh-NC, A172 sh-SNAP25, A172 Lv-NC, and A172 Lv-SNAP25 cells. We employed principal component analysis (PCA) to identify metabolic alternations between A172 Lv-NC and A172 Lv-SNAP25 cells, and each cohort separated into relative distinct clusters (**Figure 4A**). Subsequently, projections to latent structures discriminant analysis (PLS-DA) defined metabolites contributing to the greatest separation between groups. Metabolites distinguishing Lv-NC and Lv-SNAP25 (VIP > 1) were assessed and three metabolites (glutamate, glutamatic acid and L-Glutathione) played important role in this process (**Figure 4B**). Moreover, cysteine and methione metabolism, amino sugar and nucleotide sugar metabolism as well as alanine, aspartate and glutamate metabolism pathways were significantly differentiated in Lv-SNAP25 and Lv-NC tumor cells, suggesting that glutamine-related metabolism was highly activated (**Figure 4C**). Consistently, the pathway analysis showed that those involved in cysteine and methionine metabolism, amino sugar and nucleotide sugar metabolism, glutathione metabolism, alanine, aspartate and glutamate metabolism and D-glutamine and D-glutamate metabolism largely contributed to their separation (**Figure 4D**). Finally, an LC-MS assay was performed to evaluate the levels of metabolic products of glutamine metabolism (L-Aspartic acid, L-Glutamic acid and N-Acetyl-aspartic acid), which indicated a positive effect of SNAP25 in glutamate metabolic ability (**Figures 4E–G**). Consistently, the metabonomic results in sh-SNAP25 *vs* sh-NC group showed glutamate and glutathione contributed largely to their metabolic differentiation ine(**Supplementary Figures C–E** **)**. The LC-MS analysis indicated that the levels of metabolic products of glutamine metabolism (L-Aspartic acid, L-Glutamic acid and N-Acetyl-aspartic acid) in sh-SNAP25 glioma cells were significantly higher than that in sh-NC cells (**Supplementary Figures F–H** **)**. GLS catalyzes the conversion of glutamine to glutamate, acts as the rate-limiting enzyme for glutaminolysis, and exists in two isoforms, glutaminase 1 (GLS1) and 2 (GLS2) (23). Interestingly, recent findings support the function of GLS as a multifaceted protein which was not only involved in glutamate generation, but also in carcinogenesis and cancer progression as GLS2 acts as a transcriptional target of p53 and have been argued to have tumor suppressor properties, and re-expressing it in p53-deficient cells limits malignancy (24). But the exact role of GLS in SNAP25-regulated glioma progression has not been studied. By performing RT-qPCR and western blot assays, Glioma cell lines showed lower GLS expression compared to NHA cells (*p *< 0.05, **Figures 5A, B** **)**. Negative correlation between GLS expression and WHO grading was observed by immunohistochemistry assay, reflecting the similar expression pattern of GLS and SNAP25 in glioma (**Figure 5C**). Also, SNAP25-knockdown glioma cells (A172 sh-SNAP25 and U118 sh-SNAP25) showed lower GLS expression compared to sh-NC cells and overexpression of SNAP25 witnessed an increased expression of GLS according to the western blot assay (*p*<0.05, **Figure 5D**). Furthermore, the immunofluorescence assay showed consistent results (**Figure 5E**). Taken together, we demonstrated that SNAP25 contributed to boosting glutamate metabolism and it may work as a sponge to activate the rate-limiting enzyme-GLS-to make this process come true.

### SNAP25 Regulates Glioma Progression Through GLS-Mediated Glutamine Metabolism

**Figure 6:**
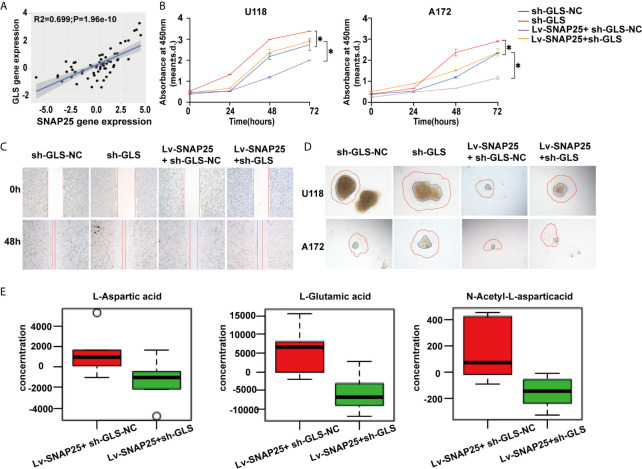
GLS is positively correlated with SNAP25. **(A)** Correlation analysis of SNAP25 and GLS by Pearson’s correlation coefficient. *P*-value is given on the figure. **(B)** CCK-8 assay of U118 and A172 glioma cells when transfected with Lv-NC, Lv-SNAP25 and/or sh-GLS-NC, sh-GLS, *p < 0.05 compared with Lv-NC or sh-GLS-NC group. **(C, D)** Wound healing assay and cancer cell spheroid invasive assay of U118 and A172 glioma cells when transfected with Lv-NC, Lv-SNAP25 and/or sh-GLS-NC, sh-GLS. **(E)** The concentration of L-Aspartic acid, L-Gluatamic acid, and N-Acetyl-L-aspartic acid from LC-MS data obtained for Lv-SNAP25 glioma cells transfected with sh-GLS-NC or sh-GLS.

**Figure 7:**
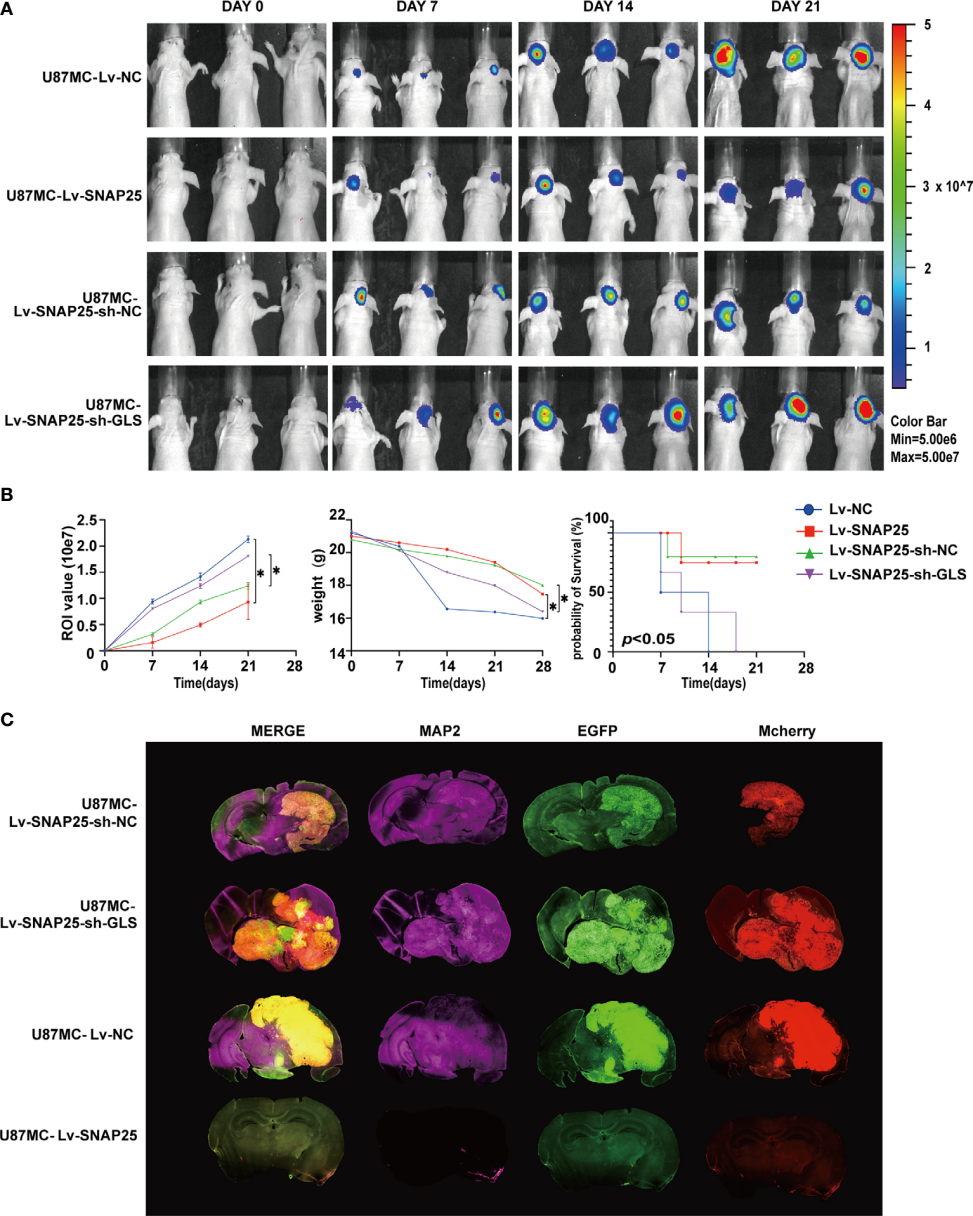
**(A)** Tumor growth was monitored over time (up to Day 21) by measuring luciferase emission (ROI). **(B)** The ROI value, weight of the mice and the probability of survival was recorded over time. *p < 0.05 compared with Lv-NC or Lv-SNAP25-sh-NC group. **(C)** Glioma cell (Mcherry staining) and neuronal dendron (MAP2 staining) in the brains of indicated mice at the time of harvesting (Day 21).

Since SNAP25 has been shown to play a vital role in glioma progression and glutamate metabolism, we next investigated the way SNAP25 functioned in this process. We hypothesized that SNAP25 could regulate GLS-mediated glutamine metabolism to inhibit glioma progression. As we have found that SNAP25 and GLS showed the concurrent expression patterns in transfected glioma cells, there was a positive correlation (R^2^ = 0.699; *p*=1.96e-10, **Figure 6A**) between SNAP25 and GLS levels in glioma tissues according to CGGA database. To further prove that SNAP25 inhibited glioma progression through activating GLS, we applied GLS-shRNA to rescue the SNAP25 overexpressed cells. The proliferation, migration, and invasion assays proved that knockdown of GLS accelerated the proliferation, migration and invasion rate of glioma parental cells, and downregulation of GLS in SNAP25-overexpressesd glioma cells could rescue the tumor-suppressive function of SNAP25 in glioma cells (**Figures 6B–D**). Importantly, knockdown of GLS in SNAP25-upregulated cells encountered a low glutamate metabolic level (**Figure 6E**). Then xenograft model of glioma *in vivo* indicated a time-dependent aggressive growth of the xenograft in rat brains and SNAP25 acted as an efficient tumor suppressor as U87Mcherry Lv-SNAP25 rats showed lower growth rate, less weight-loss and longer survival time than U87Mcherry Lv-NC animals. Furthermore, downregulating the GLS in the U87MC Lv-SNAP25 group rescued the suppressive condition (**Figures 7A, B** **)**. In the end, immunofluorenscence assay was applied to demonstrate MAP2-indicated dendritic formation and synapse plasticity levels. As is shown in **Figure 7C**, SNAP25 inhibited MAP2 expression in xenograft glioma tissues and GLS-silencing reversed this process.

The above results indicated that SNAP25 functioned as an efficient GLS sponge in glioma and SNAP25 acted as a glioma suppressor through GLS-mediated glutamine metabolism.

## Discussion

Glioma is the most devastating tumor in the central nervous system, and the exact pathogenesis of it is still unclear (25). Recent progress in molecular profiling has improved the diagnostics and classification system in which the most significant knowledge is that somatic mutations affecting the R132 residue of the isocitrate dehydrogenase 1 (IDH1) or R172 residue of the isocitrate dehydrogenase 2 (IDH2) are often detected in WHO II or III gliomas and oligodendrogliomas (26). SNAP25, a member of the SNARE family, is a membrane-binding protein in neurons that plays an indispensable role in the occurrence and development of various synaptopathies (18, 27). When comes to cancers, SNAP25 functions differently according to various cancer types. It was reported that SNAP25 was overexpressed in colon cancer samples, and abnormal expression of SNAP25 indicated a poor prognosis of colon cancer patients (21). Contradictorily, SNAP25 was identified to inhibit cancer progression as cleavage of SNAP25 could ameliorate cancer pain of a mouse melanoma model and a comprehensive bioinformatic analysis of GBM indicated that SNAP25 might act as a GBM suppressor and a biomarker in GBM treatment (28, 29). Furthermore, SNAP25 was found to have significantly lower expression levels in medulloblastoma and SNAP25 was crucial for dendrite formation which is associated with the effects of targeted chemotherapy (30). But the further mechanism of how SNAP25 regulates glioma progression remains unknown. In the present study, we first identified the expression of SNAP25 in the glioma tissues and cells. We found that SNAP25 was downexpressed in the glioma tissues and cells, and lower expression of SNAP25 showed an unfavorable prognosis in glioma patients. The effect of SNAP25 on cell proliferation, migration and invasion was also examined, and the results exhibited that SNAP25 overexpression effectively inhibited cell proliferation, migration, invasion and dendritic formation, and promoted cell glutaminolysis of glioma cells. Conversely, SNAP25 knockdown accelerated cell proliferation, migration and invasion, and decreased glutaminolysis of glioma cells. Besides, the upregulated SNAP25 could delay tumor growth and extent the overall survival time of the victims *in vivo*. The data above revealed that SNAP25 acted as an tumor suppressor in glioma and inhibited the progression of glioma.

Since SNAP25 basically functions as a critical role in synaptic activity regulating vesicle transfer between neighboring cells in the nervous system (31), SNAP25 was hypothesized to play a neuron-glioma cell interaction-activated role which can influence brain cancer growth. This represents a striking example of the core physiological function of an organ promoting the growth of cancer arising within it. An important mechanism mediating this key microenvironmental interaction is the activity-regulated degradation of SNAP25. The importance of SNAP25 in glioma pathophysiology is underscored by the finding that SNAP25 expression strongly predicts survival in human glioma and discourages expression of MAP2, an abundant microtubule-associate protein that participates in the outgrowth of neuronal processes and synaptic plasticity (32). Also, reduced expression of SNAP25 not only fails to impair synaptic transmission but instead enhance evoked glutamatergic neurotransmission potentially rely on presynaptic voltage-gated calcium channel activity (27). Taken together, these studies elucidate a fundamental dimension of the glioma microenvironment and identify a robust and targetable mechanism of SNAP25 driving glioma proliferation and progression. However, a direct influence exerted by active parenchymal neurons upon the glioma environment has not been well appreciated. The critical role of neural elements in the cancer microenvironment has recently been elucidated for prostate, pancreatic and gastric cancers, in which peripheral innervation was found to potently promote cancer progression (33, 40). Furthermore, Venkatesh et al. suggest that abundant synaptic formation plays a critical role in the microenvironment of brain tumors through the malignant hijacking of mechanisms central to brain plasticity (35).

Interestingly, a wealth of elegant data illustrates that neurotransmitters and neuropeptides can affect glioma cell behavior. Glutamate secreted from glioma cells influences their proliferation and invasion through autocrine/paracrine signaling and subsequently increases the excitability of affected cortical networks (36). Tumorigenesis requires cancer cells to increase their metabolic output to support tumor growth (16, 37). Glutamine fuels cellular bioenergetics and supports multiple biosynthetic processes, making it an important nutrient for highly proliferative cells (38). Specifically, glutamine’s carbon backbone can be utilized for the production of TCA cycle intermediates, amino acids, and other metabolites, while glutamine-derived nitrogen also promotes nucleotide biosynthesis (39–41). Specifically, the abundance of glutamine in glioma further accelerates the tumor anabolism as glutamate converted from glutamine by glutaminase may be metabolized to D2HG in IDH1 mutated glioma cells accompanied by a loss of proper enzymatic activity (26). The proper function of glutaminase would be an important factor between the IDH1/2 mutational status and the WHO grade classification of gliomas.

In this study, the metabolic activity of glioma cells showed a significant discrepancy between SNAP25 overexpression and SNAP25 control cell groups and glutathione metabolism, alanine, aspartate and glutamate metabolism largely contributed to their separation. Moreover, glutamine-related metabolism was highly facilitated as increased L-aspartate acid, L-glutamate acid and N-acetyl-L-aspartic acid abundance was observed. Glutamatergic systems govern neuron-glia and glia-glia interactions and coordinate metabolic coupling of local cells (42, 43). Activation of the glutamine metabolism maintains synaptic homeostasis and regulates synaptic formation/plasticity. GLS, which deaminates glutamine to glutamate, reduces proliferation and tumorigenicity in certain cancer models (44). Conversely, GLS2 can be induced by the tumor suppressor p53 as a tumor suppressor (45). We confirmed that GLS acted as a metabolic target of SNAP25 and consequently decelerated glioma progression. The correlation between SNAP25 and GLS is positive and the correlation coefficient is 0.699 (*p*=1.96e-10). Then further validation was applied in glioma tissues and cells and poor GLS expression was found in gliomas compared to normal tissues. Finally, knockdown of GLS could rescue the antitumor effect of SNAP25 *in vitro* and *in vivo*.

In summary, the present study is the first to investigate synapse-related gene expression patterns in glioma patients and identify their relationship to patient outcome. The synaptic signature SNAP25 identified in our study exhibited potential as a biomarker of OS in glioma patients and indicated a relationship between neuron-glioma cell interaction and glioma progression. But the study just proved the positive correlation between SNAP25 and GLS, the cause effect of SNAP25 in regulation of GLS expression and the detail regulative mechanism was not figured out. Also, the relationship between SNAP25 and MAP2 was not well distinguished as the low expression of MAP2 in U87Mcherry Lv-SNAP25 glioma cell-transplanted-xenograft may be simply related to the lack of tumor growth and size.

Understanding the microenvironmental mechanisms underlying synapse plasticity and its effect on tumor prognosis can provide insights into the identification of diagnostic and therapeutic targets for glioma.

## Data Availability Statement

The original contributions presented in the study are publicly available. This data can be found here: www.ebi.ac.uk/metabolights/MTBLS2806 (Identifier MTBLS2806).

## Ethics Statement

The studies involving human participants were reviewed and approved by the ethical committee of Zhujiang Hospital. The patients/participants provided their written informed consent to participate in this study. The animal study was reviewed and approved by the ethical committee of Zhujiang Hospital.

## Author Contributions

QH and CL: conception and design, experiments conduction, data analysis, and manuscript writing. YD, QH, and CL: experiments conduction and data analysis. ZH, BL, HZ, and NX: final approval of manuscript. HG: conception and design, and final approval of manuscript. All authors contributed to the article and approved the submitted version.

## Funding

This study was supported by funds from the National Science Foundation of Guangdong Province (2017A030308001), Guangdong Province Science and Technology Innovation Strategy Special Fund (2018A030310422), the Guangdong Medical Science and Technology Research Fund (A2018542), and the National Nature Science Foundation of China (81874019, 82073193).

## Conflict of Interest

The authors declare that the research was conducted in the absence of any commercial or financial relationships that could be construed as a potential conflict of interest.

## Publisher’s Note

All claims expressed in this article are solely those of the authors and do not necessarily represent those of their affiliated organizations, or those of the publisher, the editors and the reviewers. Any product that may be evaluated in this article, or claim that may be made by its manufacturer, is not guaranteed or endorsed by the publisher.

## Supplementary Material

Supplementary Figure 1 **(A, B)** Knockdown and over-expression of endogenous SNAP25 in specific shRNA transduced glioma cells by RT-qPCR and western blot. **p *< 0.05 compared with the sh-NC cells. **(C)** Score plot of the principle component analysis results for LC-MS data obtained for sh-NC and sh-SNAP25 glioma cells. **(D)** Loading plots of the principle component analysis results for LC-MS data obtained for sh-NC and sh-SNAP25 glioma cells. Metabolites 261 (glutamate) and 262 (glutamic acid) contributed largely to their separation. **(E)** Pathway analysis results for LC-MS data obtained for sh-NC and sh-SNAP25 glioma cells. Alanine, aspartate and glutamate metabolism pathway shows a high pathway plotimpact. **(F-H)** The concentrantion of L-Aspartic acid **(E)**, L-Gluatamic acid **(F)**, and N-Acetyl-L-aspartic acid.
