# Supplementary figures and images for: SNAP25 Inhibits Glioma Progression by Regulating Synapse Plasticity via GLS-Mediated Glutaminolysis
Source: Front Oncol. 2021 Aug 16;11:698835. doi: 10.3389/fonc.2021.698835 (PMC8416623; doi:10.3389/fonc.2021.698835)

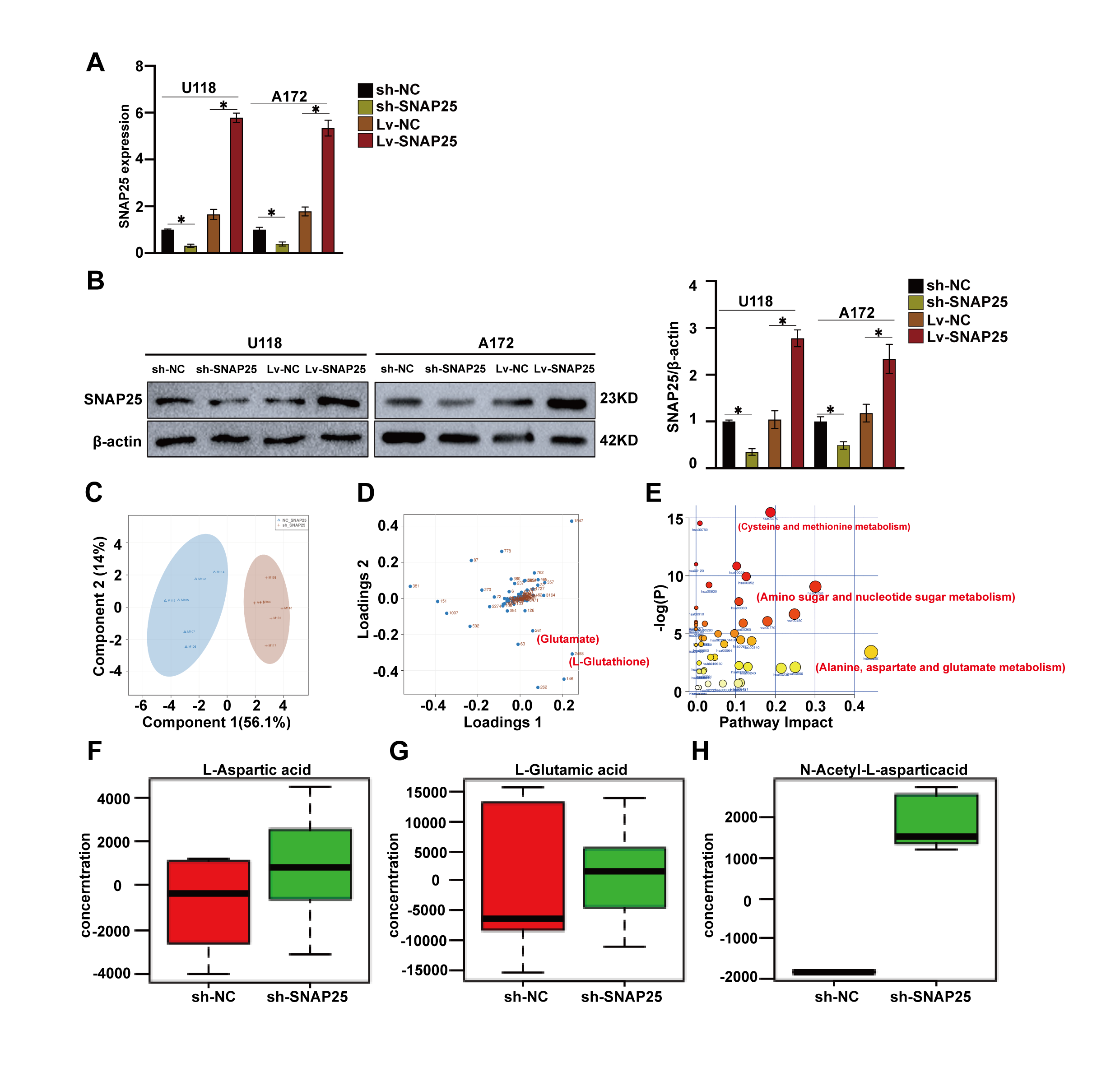

Supplement: Supplementary Figure 1 — (A, B) Knockdown and over-expression of endogenous SNAP25 in specific shRNA transduced glioma cells by RT-qPCR and western blot. *p < 0.05 compared with the sh-NC cells. (C) Score plot of the principle component analysis results for LC-MS data obtained for sh-NC and sh-SNAP25 glioma cells. (D) Loading plots of the principle component analysis results for LC-MS data obtained for sh-NC and sh-SNAP25 glioma cells. Metabolites 261 (glutamate) and 262 (glutamic acid) contributed largely to their separation. (E) Pathway analysis results for LC-MS data obtained for sh-NC and sh-SNAP25 glioma cells. Alanine, aspartate and glutamate metabolism pathway shows a high pathway plotimpact. (F-H) The concentrantion of L-Aspartic acid (E), L-Gluatamic acid (F), and N-Acetyl-L-aspartic acid. [file Image_1.tif]
