# Supplementary material for: SNAP25 Inhibits Glioma Progression by Regulating Synapse Plasticity via GLS-Mediated Glutaminolysis
Source: Front Oncol. 2021 Aug 16;11:698835. doi: 10.3389/fonc.2021.698835 (PMC8416623; doi:10.3389/fonc.2021.698835)
Supplement: Supplementary file 2 [file DataSheet_1.pdf]

Supplementary Table 1: Association between SNAP25 expression levels and clinical/pathological characteristics in 40 patients with glioma

Characteristics	No. of patients （n=40）	Low expression (n=23)	High expression (n=17)	*P*
Gender				
Male	21 (52.5%)	12（52.2%）	9（53.0%）	0.642
Female	19 (47.5%)	11（47.8%）	8（47.0%）	
Age, year				
<50	8 (20.0%)	3（13.0%）	4（23.5%）	0.504
≥50	32 (80.0%)	20（87.0%）	20（76.5%）	
WHO Grade				
I/II	9 (22.5%)	1（4.0%）	9（53.0%）	<0.001*
III/IV	31 (77.6%)	22（96.0%）	8（47.0%）	

Abbreviation: WHO=World Health Organization.

Statistical significance (p<0.001).

Supplementary Table 2：Primer sequences used for PCR

mRNA	Forward Primer	Reverse Primer
GAPDH	GGAGCGAGATCCCTCCAAAAT	GGCTGTTGTCATACTTCTCATGG
SNAP25	ACCAGTTGGCTGATGAGTCG	CAAAGTCCTGATACCAGCATCTT
GLS	AGGGTCTGTTACCTAGCTTGG	ACGTTCGCAATCCTGTAGATTT

Supplementary Table 3：Abbreviations and their full names

ABBREVIATIONS	THE FULL NAMES
SNAREs	soluble N-ethylmaleimide-sensitive factor attachment protein receptors
SNAP25	synaptosomal-associated protein 25
TCGA	The Cancer Genome Atlas
CGGA	the Chinese Glioma Genome Atlas
GSEA	gene set enrichment analysis
LGG	low grade glioma
GBM	glioblastoma
LC-MS	Liquid chromatography-high resolution mass spectrometry
MAP2	Microtubule Associated Protein 2
GLS	glutaminase

TCA	tricarboxylic acid
U87MC	U87MG-mCherry cell line
DEGS	differentially expressed genes
OS	overall survival
PCA	principal component analysis
PLS-DA	projections to latent structures discriminant analysis
